# Anlotinib in patients with medullary thyroid carcinoma with negative prognostic factors: A sub-analysis based on the ALTER01031 study

**DOI:** 10.3389/fonc.2022.852032

**Published:** 2022-11-22

**Authors:** Jingzhu Zhao, Yihebali Chi, Chuanxiang Hu, Xiaohong Chen, Minghua Ge, Yuan Zhang, Zhuming Guo, Jun Wang, Jie Chen, Jiewu Zhang, Ying Cheng, Zhendong Li, Hui Liu, Jianwu Qin, Jingqiang Zhu, Ruochuan Cheng, Zhengang Xu, Dapeng Li, Pingzhang Tang, Ming Gao, Xiangqian Zheng

**Affiliations:** ^1^ Department of Thyroid and Neck Oncology, Tianjin Medical University Cancer Institute and Hospital, National Clinical Research Center for Cancer; Key Laboratory of Cancer Prevention and Therapy, Tianjin’s Clinical Research Center for Cancer, Tianjin, China; ^2^ Department of Medical Oncology, National Cancer Center/National Clinical Research Center for Cancer/Cancer Hospital, Chinese Academy of Medical Sciences and Peking Union Medical College, Beijing, China; ^3^ Department of Otolaryngology Head and Neck Surgery, Beijing Tongren Hospital, Capital Medical University / Key Laboratory of Otolaryngology Head and Neck Surgery, Ministry of Education, Beijing Institute of Otolaryngology, Beijing, China; ^4^ Head and Neck Surgery, Institute of Cancer and Basic Medicine (ICBM), Chinese Academy of Sciences; Cancer Hospital of the University of Chinese Academy of Sciences; Zhejiang Cancer Hospital, Hangzhou, China; ^5^ Department of Head, Neck and Thyroid Surgery, Zhejiang Provincial People’s Hospital, People’s Hospital of Hangzhou Medical College, Hangzhou, China; ^6^ Head and Neck Surgery (Department), Jiangsu Cancer Hospital (Jiangsu Institute of Cancer Research, Nanjing Medical University Affiliated Cancer Hospital), Nanjing, China; ^7^ Head and Neck Surgery, Sun Yat-sen University Cancer Center, Guangzhou, China; ^8^ Head and Neck Surgery, Gansu Provincial Cancer Hospital, Lanzhou, China; ^9^ Head and Neck Surgery Department I, Hunan Cancer Hospital, Changsha, China; ^10^ Thyroid Surgery Ward, Harbin Medical University Cancer Hospital, Harbin, China; ^11^ Department of Medical Oncology, Jilin Cancer Hospital, Changchun, China; ^12^ Head and Neck Department, Tumor Hospital of China Medical University, Liaoning Tumor Hospital & Institute, Shenyang, China; ^13^ Head and Neck Surgery, Fujian Cancer Hospital, Fuzhou, China; ^14^ Thyroid & Head and Neck Surgery, Henan Cancer Hospital, Affiliated Cancer Hospital of Zhengzhou University, Zhengzhou, China; ^15^ Thyroid Surgery, West China Hospital, Sichuan University, Chengdu, China; ^16^ Department of Thyroid Surgery, First Affiliated Hospital of Kunming Medical University, Kunming, China; ^17^ Head and Neck Surgery, National Cancer Center/National Clinical Research Center for Cancer/Cancer Hospital, Chinese Academy of Medical Sciences and Peking Union Medical College, Beijing, China; ^18^ Department for VIP, National Cancer Center/National Clinical Research Center for Cancer/Cancer Hospital, Chinese Academy of Medical Sciences and Peking Union Medical College, Beijing, China; ^19^ Breast & Thyroid Surgery, Tianjin Union Medical Center, Tianjin, China

**Keywords:** medullary thyroid carcinoma, anlotinib, subgroup analysis, older age, bone metastases

## Abstract

**Background:**

Medullary thyroid carcinoma (MTC) is a rare type of thyroid cancer; however, it accounted for 13.4% of the disease-specific mortalities. ALTER01031 (NCT02586350) was a randomised, placebo-controlled phase 2b trial that evaluated the efficacy and safety of anlotinib in locally advanced or metastatic MTC. This *post hoc* analysis aimed to evaluate the efficacy and safety of anlotinib in older patients and those with bone metastases using ALTER01031.

**Methods:**

In ALTER01031, anlotinib significantly prolonged the median progression-free survival (PFS) from 11.1 months to 20.7 months compared with placebo in the whole population. Patients who were older (≥ 50 years) or had bone metastases were selected. PFS and overall survival (OS) were estimated and compared between patients receiving anlotinib or placebo in each subgroup. A sub-analysis of tumour response and safety was also performed.

**Results:**

Patients with older age or bone metastases experienced rapid disease progression as the median PFS was 6.8 months and 7.0 months respectively in the placebo group. Anlotinib significantly improved the median PFS to 17.5 months (*P* = 0.002) and 20.7 months (*P* = 0.029) with hazard ratio (HR) of 0.31 (95% CI, 0.15–0.68) and 0.44 (95% CI, 0.20–0.94) compared with placebo. Significant benefit in OS was observed in patients with older age after a longer follow-up (HR = 0.47 [95% CI, 0.22–0.99], *P* = 0.041). The safety profile of these subgroups was similar to that of the entire population.

**Conclusion:**

This sub-analysis demonstrated significant survival benefits and favourable safety of anlotinib in patients with MTC who had old age or bone metastases, supporting the feasibility of anlotinib in these patients.

## Introduction

Medullary thyroid carcinoma (MTC) is a rare tumour that originates from C cells, accounts for only 2% of all malignant thyroid neoplasms (1) and is responsible for more than one-tenth of deaths caused by thyroid cancer (2). MTC is insensitive to chemotherapy (3) and has a low tumour mutation burden (4). Angioinvasion is highly related to the recurrence and metastasis of MTC (5) and multi-kinase inhibitors with anti-angiogenic activity, such as cabozantinib and vandetanib, are standard treatments for advanced MTC (6, 7).

Treatment with multi-kinase inhibitors (MKIs) against angiogenesis significantly prolongs the progression-free survival (PFS) of patients with unresectable lesions, although limitations still exist. For example, the high incidence of grade 3 or worse QT interval prolongation limits the application of vandetanib in patients with heart conduction disorders (7). In EXAM, 3.3% patients in cabozantinib group experienced grade 3 or worse treatment-related bleeding and 79% could not tolerate the standard dose continuously (6). Selpercatinib and pralsetinib have shown favourable efficacy however, these highly selective inhibitors are only suitable for patients with RET mutations (8, 9).

Anlotinib is a multi-kinase inhibitor that targets both angiogenesis and tumour cell proliferation by blocking vascular endothelial growth factor (VEGFR), platelet-derived growth factor receptor, fibroblast growth factor receptor, and c-Kit (10). Anlotinib has demonstrated promising efficacy and safety in the treatment of locally advanced or metastatic MTC in a phase 2b study (NCT02586350, ALTER01031), which was the first randomised controlled study of MTC with a relatively larger sample size after the approval of vandetanib and cabozantinib. especially in the Asian population (11).

Generally, older age and bone metastases have been identified as negative prognostic factors of MTC (12). In a retrospective study, the incidence of bone metastases in patients with MTC was 19% (13), higher than in the 3.9% of all types of thyroid cancer (14). In the presence of bone events, overall and disease-specific mortality of patients with MTC was higher with hazard ratio of 1.53 and 2.47 (13). Here, we report the results of a *post hoc* analysis of ALTER01031 to validate the efficacy and safety of anlotinib in older patients and those with bone metastases and further investigate the feasibility of anlotinib in these populations.

## Materials and methods

### Study design and participants

This analysis assessed subgroups of patients with older age and bone metastasis from ALTER01031, which was a randomized, placebo-controlled, double blind, phase 2b study (NCT02586350). The details of ALTER01031 have been disclosed previously (11). Patients eligible for ALTER01031 were 18–70 years old, pathologically confirmed unresectable locally advanced or metastatic MTC, and had at least one measurable lesion according to RECIST 1.1. Other key eligibility criteria included an Eastern Cooperative Oncology Group (ECOG) performance status (PS) score of 0–1 and previous anlotinib or other VEGFR TKI naïve. This study was conducted in accordance with the Declaration of Helsinki and approved by the Institutional Review Board. Written informed consent was obtained from all patients. Patients were randomly assigned in a 2: 1 ratio to receive anlotinib at a dose of 12 mg once daily for 14 days in a 3-week cycle or placebo. The patients in the placebo group were unblinded and received open-label anlotinib after disease progression.

In a previous analysis of MTC, compared to patients younger than 50 years, those older than 50 years had an increased risk of cancer-specific mortality (15). Therefore, 50 years was chosen as the cut-off age. The outcomes of older patients (≥ 50 years old) and those with bone metastases were analysed.

### Endpoints

The primary endpoint of interest for this analysis was PFS, defined as the time from randomisation to disease progression or death. Secondary endpoints included objective response rate (ORR), overall survival (OS), safety, and change in calcitonin levels. Tumour response was determined based on radiographic images by an independent review committee. Tumour response was evaluated and confirmed based on computed tomography by an independent review committee according to RECIST 1.1, every 2 treatment cycles for the first 12 cycles, then every 4 cycles. Blood samples were collected from patients under blinded treatment at baseline and at the end of cycles 1, 2, 4, and 8 to evaluate the change in serum calcitonin level. The occurrence and severity of adverse events (AEs) were assessed according to the Common Toxicity Standards of the National Cancer Institute (CTC AE 4.0).

### Statistical analysis

PFS and OS were estimated and plotted using the Kaplan–Meier method and compared using the log-rank test between subgroups. The hazard ratio (HR) was assessed using the Cox proportional hazards model. Rank-preserving structural failure time (RPSFT) models were used to adjust the potential bias from the crossover treatment for patients in the placebo group, and the adjusted OS and HR will also be reported. The ORR within each subgroup was calculated as the percentage of patients with complete and partial responses. The AEs and incidence in each subgroup are listed in the table. The Wilcoxon signed-rank test was used to compare CEA and calcitonin levels between the treatment arms. All statistical analyses were performed using SAS version 9.2.

## Results

### Patients and overall efficacy

A total of 91 patients were enrolled: 62 in the anlotinib group and 29 in the placebo group. At the data cut-off date on September 30, 2018, the study satisfied its primary endpoint that the median PFS reached 20.7 months (95% CI, 14.0–34.6) in the anlotinib group, significantly longer than that of 11.1 months (95% CI, 5.8–14.3) in the placebo group (HR = 0.53 (95% CI, 0.30–0.95), *P* = 0.029). OS data was immature, and the HR was 0.92 (95% CI, 0.43–1.97, *P* = 0.826). After the adjustment with RPSFT model to modify the potential bias due to crossover, the HR became 0.73 (95% CI, 0.39–1.56; *P* = 0.410), indicating the trend of OS benefit with anlotinib. The ORR and disease control rate (DCR) for the anlotinib treatment for the entire population were 48.4% and 88.7%, respectively.

### Efficacy for subgroups

Fifty-one patients older than 50 years were enrolled, 36 in the anlotinib group and 15 in the placebo group. The baseline characteristics were similar between the two subgroups in terms of sex, age, treatment history, and metastatic sites (**Table 1**).

**Table 1:** Baseline characteristics of patients in the different subgroups.

	Patients older than 50 years		Patients with bone metastasis	
	Anlotinib (n=36)	Placebo (n=15)	Anlotinib (n=29)	Placebo (n=18)
Age (years), n(%)				
Median Range	58.2 53.5-63.3	58.5 54.3-64.3	53.9 49.5-59.2	56.1 44.1-61.0
Sex, n(%)				
Male Female	23 (63.9) 13 (36.1)	11 (73.3) 4 (26.7)	20 (69.0) 9 (31.0)	9 (50.0) 9 (50.0)
ECOG PS, n(%)				
0 1	9 (25.0) 25 (75.0)	4 (26.7) 11 (73.3)	11 (37.9) 18 (62.1)	6 (33.3) 12 (66.7)
Surgery history, n(%)				
Yes No	33 (91.7) 3 (8.3)	12 (80.0) 3 (20.0)	28 (96.5) 1 (3.5)	16 (88.9) 2 (11.1)
Radiotherapy history, n(%)				
Yes No	12 (33.3) 24 (66.7)	4 (26.7) 11 (73.3)	11 (37.9) 18 (62.1)	4 (22.2) 14 (77.8)
Chemotherapy history, n(%)				
Yes No	5 (13.9) 31 (86.1)	1 (6.7) 14 (93.3)	4 (13.8) 25 (86.2)	3 (16.7) 15 (83.3)
Stage of disease, n(%)				
IVA IVB IVC	3 (8.3) 1 (2.8) 32 (88.9)	1 (6.7) 0 (0.0) 14 (93.3)	1 (3.5) 1 (3.5) 27 (93.1)	1 (5.6) 0 (0.0) 17 (94.4)
Liver				
Yes No	19 (52.8) 17 (47.2)	8 (53.3) 7 (46.7)	18 (62.1) 11 (37.9)	13 (72.2) 5 (27.8)
Lung metastasis, n(%)				
Yes No	23 (63.9) 13 (36.1)	11(73.3) 4 (26.7)	17 (58.6) 12 (41.4)	13 (72.2) 5 (27.8)
Lymph metastasis, n(%)				
Yes No	32 (88.9) 4 (11.1)	14 (93.3) 1 (6.7)	26 (89.7) 3 (10.3)	16 (88.9) 2 (11.1)
Bone metastasis, n(%)				
Yes No	19 (52.8) 17 (47.2)	10 (66.7) 5 (33.3)	29 (100.0) 0 (0.0)	18 (100.0) 0 (0.0)

In the whole population, multivariate Cox regression analysis including factors of age, treatment, ECOG PS score, and disease progression within 12 months before enrolment indicated that older age was an independent risk factor for disease progression (HR = 1.89 [95% CI 1.01–3.54]; *P* = 0.046) and death (HR = 4.07 [95% CI, 1.54–10.80]; *P* = 0.005). In addition, for patients who received placebo, the median PFS and OS for those older than 50 years were 6.8 and 18.5 months, showing a significantly higher risk of disease progression and death than those younger than 50 years, with an HR of 3.29 (95% CI, 1.25–8.66) and 10.46 (95% CI, 1.32–82.84) for PFS (*P* = 0.011) and OS (*P* = 0.006) respectively.

**Figure 1:**
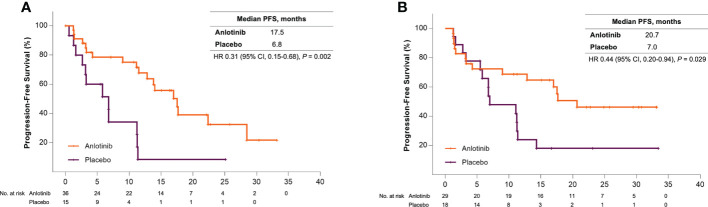
**(A)** Kaplan-Meier curves of progression-free survival for patients older than 50 years received anlotinib (orange) or placebo (violet). **(B)** Kaplan-Meier curves of progression-free survival for patients with bone metastases received anlotinib (orange) or placebo (violet).

**Figure 2:**
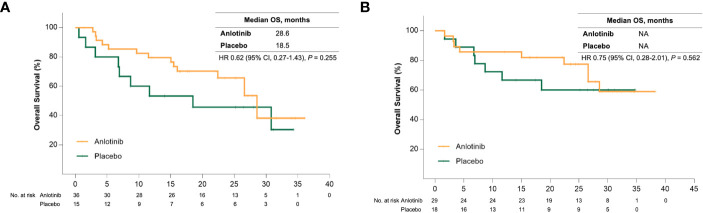
**(A)** Kaplan-Meier curves of overall survival for patients older than 50 years received anlotinib (orange) or placebo (green). **(B)** Kaplan-Meier curves of overall survival for patients with bone metastases received anlotinib (orange) or placebo (green).

**Figure 3:**
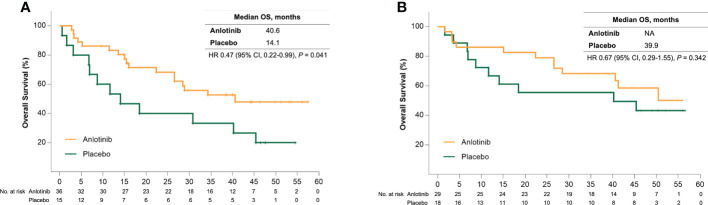
**(A)** Kaplan-Meier curves of overall survival for patients older than 50 years received anlotinib (orange) or placebo (green) after extension of follow up. **(B)** Kaplan-Meier curves of overall survival for patients with bone metastases received anlotinib (orange) or placebo (green) after extension of follow up.

Sub-analysis showed a significant survival benefit from anlotinib for patients older than 50 years. At the data cut-off date, 18 (50%) and 12 (80%) patients in the anlotinib and placebo group had disease progression, respectively, and the median PFS was 17.5 months versus 6.8 months (HR = 0.31 [95% CI, 0.15–0.68], *P* = 0.002) (**Figure 1A**). Seven patients (46.7%) in the placebo group received open-label anlotinib after disease progression. At the primary analysis, 15 (41.7%) and 9 (60%) older patients in the anlotinib and placebo groups died and the median OS was prolonged after anlotinib treatment without statistical significance (28.6 months vs. 18.5 months, HR = 0.62 (95% CI, 0.27–1.43), *P* = 0.255) (**Figure 2A**). The trend of the OS benefit from anlotinib treatment became more obvious as time progressed. After two more years of added follow-up, 17 (47.2%) and 12 (80%) older patients in the placebo and anlotinib groups had died, respectively, and the OS benefit from anlotinib treatment achieved statistical significance (40.6 months vs 14.1 months, HR = 0.470 (95% CI, 0.22–0.99), *P* = 0.041) (**Figure 3A**).

Bone metastases are another crucial poor prognostic factor in patients with MTC. In the ALTER01031 study, 47 of 91 patients had bone metastases at baseline: 29 in the anlotinib group and 18 in the placebo group. Baseline characteristics were also similar between the two subgroups (**Table 1**). In accordance with our prospection, bone metastases led to a rapid disease progression with a median PFS of only 7.0 months for patients in the placebo group, which was shorter than the 11.1 months for the whole population. At the time of the primary analysis, 14 (48.3%) and 14 (77.8%) patients in the anlotinib and placebo groups had disease progression respectively. Anlotinib significantly prolonged the median PFS to 20.7 months and decreased the risk of disease progression by 56% (HR = 0.44 (95% CI, 0.20–0.94), *P* = 0.029) (**Figure 1B**). The median OS was not reached in either group, with an estimated HR of 0.75 (95% CI, 0.28–2.01, *P* = 0.562) (**Figure 2B**). At the second analysis after two more years of follow-up, 12 (41.4%) and 10 (55.6%) patients with bone metastases in the two groups died, and the trend of OS benefit from anlotinib treatment also extended as that the median OS was not achieved versus 39.9 months (HR = 0.67, [95% CI, 0.29–1.55], *P* = 0.342) (**Figure 3B**).

For patients treated with anlotinib, the ORR was 47.2% and 37.9% for older age and bone metastasis subgroups, slightly weaker than those in the entire population (ORR, 48.4%) (11). The median duration of response was 15.6 months and not reached respectively. A significant decrease in serum calcitonin levels was observed across the two anlotinib treatment arms. In the 50 older patients who underwent assessment of serum calcitonin and CEA levels at baseline and after two treatment cycles, the median calcitonin and CEA level decreased significantly in the anlotinib arm, from 7576 ng/L to 1803 ng/L (*P* < 0.0001), 46 ng/mL to 27 ng/mL (*P=* 0.004), respectively. In the placebo arm, no obvious change in the median calcitonin and CEA level was observed (from 24871 ng/L to 24160 ng/L) (*P* = 0.895), (from 108 ng/mL to 110 ng/mL) (*P* = 0.363), respectively. Similarly, for patients with bone metastasis, the median calcitonin and CEA level decreased significantly in the anlotinib arm, from 14306 ng/L to 7798 ng/L (*P* < 0.0001), 129 ng/mL to 101 ng/mL (*P=* 0.003), respectively. While in the placebo arm, the median calcitonin and CEA was even slightly increased from 8708.5 ng/L to 9220.5 ng/L (*P* = 0.918), 105 ng/mL to 115 ng/mL, *P* = 0.201), respectively.

### Safety

In the entire population, the incidence of treatment-related adverse events (TRAEs) was 100% and 89.7% in the anlotinib and placebo groups, respectively. The serious TRAEs occurred in 12.9% of patients who received anlotinib. The incidence of dose reduction for patients in the anlotinib group was 32.3%.

In patients with older age or bone metastases, the incidence and types of TRAEs were similar to those in the entire population. In patients older than 50 years, the incidence of TRAEs was 100% and 86.7% in the anlotinib and placebo groups, respectively (*P* = 0.082) (**Table 2**). The incidence of grade 3 or worse TRAEs was also similar between the two treatment arms (55.6% vs. 53.3%, *P* = 0.88). The most common TRAEs for patients with older age included hypertension (58.3% vs. 33.3%), palmar-plantar erythrodysesthesia syndrome (55.6% vs. 20.0%), and proteinuria (50.0% vs. 40.0%). Six (16.7%) patients in the anlotinib arm experienced serious TRAE, while none was observed in the placebo arm. Permanent withdrawal of anlotinib caused by TRAEs occurred in five (13.9%) patients with older age.

**Table 2:** Common treatment-related adverse events (≥20%) occurred in patients older than 50 years.

	All grades		Grade 3 or worse	
Adverse events n(%)	Anlotinib (n=36)	Placebo (n=15)	Anlotinib (n=36)	Placebo (n=15)
**All**	36 (100.0)	13 (86.7)	20 (55.6)	8 (53.3)
**Hypertension**	21 (58.3)	5 (33.3)	5 (13.9)	2 (13.3)
Palmar-plantar erythrodysesthesia syndrome	20 (55.6)	3 (20.0)	6 (16.7)	0 (0.0)
**Proteinuria**	18 (50.0)	6 (40.0)	0 (0.0)	0 (0.0)
**Hypertriglyceridemia**	18 (50.0)	5 (33.3)	3 (8.3)	1 (6.7)
**QT interval prolongation**	14 (38.9)	2 (13.3)	0 (0.0)	0 (0.0)
**Diarrhea**	11 (30.6)	3 (20.0)	1 (2.8)	0 (0.0)
**Fatigue**	11 (30.6)	3 (20.0)	1 (2.8)	0 (0.0)
**Hypercholesteremia**	10 (27.8)	4 (26.7)	0 (0.0)	0 (0.0)
**GGT elevation**	10 (27.8)	2 (13.3)	1 (2.8)	0 (0.0)
**Lipase elevation**	9 (25.0)	4 (26.7)	4 (11.1)	3 (20.0)
**Urine erythrocyte positive**	9 (25.0)	3 (20.0)	0 (0.0)	0 (0.0)
**ALT increase**	9 (25.0)	2 (13.3)	0 (0.0)	0 (0.0)
**LDL increase**	9 (25.0)	1 (6.7)	0 (0.0)	0 (0.0)
**Hoarseness**	9 (25.0)	0 (0.0)	0 (0.0)	0 (0.0)
**AST increase**	8 (22.2)	2 (13.3)	1 (2.8)	0 (0.0)
**Amylase elevation**	7 (19.4)	6 (40.0)	3 (8.3)	3 (20.0)
**Conjugated bilirubin increase**	7 (19.4)	2 (13.3)	1 (2.8)	0 (0.0)
**Platelet count decrease**	6 (16.7)	2 (13.3)	1 (2.8)	1 (6.7)
**Pharyngalgia**	6 (16.7)	1 (6.7)	2 (5.6)	1 (6.7)
**Bilirubin increase**	6 (16.7)	0 (0.0)	0 (0.0)	0 (0.0)
**LDH increase**	6 (16.7)	0 (0.0)	1 (2.8)	0 (0.0)
**Neutrophil count decrease**	5 (13.9)	3 (20.0)	0 (0.0)	0 (0.0)
**Anorexia**	5 (13.9)	3 (20.0)	1 (2.8)	0 (0.0)

All patients with bone metastases in the anlotinib and placebo arms experienced at least one TRAE (**Table 3**). The most common TRAEs were palmar-plantar erythrodysesthesia syndrome (65.5% vs. 38.9%), proteinuria (62.1% vs. 38.9%), and hypertension (55.2% vs. 33.3%). The incidence of grade 3 or worse TRAEs was 58.6% and 50% in the anlotinib and placebo arms, respectively (*P* = 0.56). Treatment-related severe adverse events (SAEs) occurred in 17.2% and 5.6% patients with bone metastases in two treatment arms (*P* = 0.38). Permanent withdrawal of anlotinib caused by TRAEs occurred in three of 29 patients (10.3%).

**Table 3:** Common treatment-related adverse events (≥20%) occurred in patients with bone metastases.

	All grades		Grade 3 or worse	
Adverse events n(%)	Anlotinib (n=29)	Placebo (n=18)	Anlotinib (n=29)	Placebo (n=18)
**All**	29 (100.0)	18 (100.0)	17 (58.6)	9 (50.0)
Palmar-plantar erythrodysesthesia syndrome	19 (65.5)	7 (38.9)	4 (13.8)	2 (11.1)
**Proteinuria**	18 (62.1)	7 (38.9)	0 (0.0)	0 (0.0)
**Hypertension**	16 (55.2)	6 (33.3)	1 (3.5)	2 (11.1)
**QT interval prolongation**	15 (51.7)	2 (11.1)	0 (0.0)	0 (0.0)
**Diarrhea**	13 (44.8)	8 (44.4)	2 (6.9)	0 (0.0)
**Hypertriglyceridemia**	12 (41.4)	7 (38.9)	2 (6.9)	0 (0.0)
**Fatigue**	12 (41.4)	5 (27.8)	2 (6.9)	0 (0.0)
**Hypercholesteremia**	11 (37.9)	4 (22.2)	0 (0.0)	1 (5.6)
**ALT increase**	11 (37.9)	1 (5.6)	1 (3.5)	0 (0.0)
**Pharyngalgia**	10 (34.5)	4 (22.2)	1 (3.5)	1 (5.6)
**Urine erythrocyte positive**	10 (34.5)	4 (22.2)	0 (0.0)	0 (0.0)
**GGT elevation**	10 (34.5)	1 (5.6)	1 (3.5)	0 (0.0)
**LDL increase**	10 (34.5)	1 (5.6)	0 (0.0)	0 (0.0)
**Hoarseness**	10 (34.5)	0 (0.0)	0 (0.0)	0 (0.0)
**Lipase elevation**	9 (31.0)	5 (27.8)	3 (10.3)	2 (11.1)
**Anorexia**	8 (27.6)	5 (27.8)	1 (3.5)	0 (0.0)
**AST increase**	8 (27.6)	2 (11.1)	0 (0.0)	0 (0.0)
**Conjugated bilirubin increase**	7 (24.1)	1 (5.6)	1 (3.5)	0 (0.0)
**Lipase elevation**	6 (20.7)	6 (33.3)	2 (6.9)	2 (11.1)
**Platelet count decrease**	6 (20.7)	1 (5.6)	1 (3.5)	0 (0.0)
**LDH increase**	6 (20.7)	0 (0.0)	1 (3.5)	0 (0.0)
**Bilirubin increase**	5 (17.2)	2 (11.1)	0 (0.0)	0 (0.0)
**Neutrophil count decrease**	5 (17.2)	2 (11.1)	0 (0.0)	0 (0.0)
**Back pain**	5 (17.2)	1 (5.6)	0 (0.0)	0 (0.0)
**White blood cell count decrease**	4 (13.8)	3 (16.7)	0 (0.0)	0 (0.0)
**Toothache**	4 (13.8)	3 (16.7)	0 (0.0)	0 (0.0)

GGT γ-Glutamyltransferase, ALT alanine aminotransferase, AST aspartate aminotransferase, LDH lactate dehydrogenase.

Grade 3 or worse Q-T interval prolongation was not observed in either subgroup. One patient with bone metastases experienced grade 3 haematuria. Fatal TRAEs occurred in two cases. One patient older than 50 years had persistent grade 3 hepatic dysfunction and grade 3 hyponatraemia who eventually died during hospitalisation. Another patient aged > 50 year who had bone metastasis manifested with thrombocytopenia after the 4^th^ cycles, for the response was evaluated as PR, he did not comply with the demand of treatment discontinuation by the investigator and repeatedly refused to discontinue treatment. He eventually died due to the complications caused by thrombocytopenia.

## Discussion

Research on the treatment efficacy of MKIs in patients with MTC who were older or had bone metastases is lacking. Subgroup analysis for these populations was not reported in the study of vandetanib but was only presented in a forest plot in the study of cabozantinib (6, 7). To our knowledge, this is the first systematic evaluation of the efficacy of MKIs in these subpopulations.

Fifty years was selected as the cut-off value to define older patients in our sub-analysis. The *post-hoc* analysis focusing on patients of different ages in the placebo group indicated that age > 50 years was significantly related with a high risk of disease progression and death (HR: 3.29 [95% CI, 1.25–8.66]) for PFS and 10.46 (95% CI, 1.32–82.84) for OS. Although the subgroups were not prescribed, the baseline characteristics were mainly balanced between older patients in the different treatment arms. Anlotinib significantly improved the PFS for older patients from 6.8 months to 17.5 months and the HR was 0.31. Importantly, anlotinib treatment resulted in a significant long-term OS benefit. After a median follow-up of approximately 4 years, anlotinib treatment significantly decreased the mortality risk of MTC by 53% (HR, 0.470 [95% CI, 0.224–0.986], *P* = 0.041). To our knowledge, this is the first report of a definite OS benefit in older patients with MTC, although the results should be interpreted with caution in a subgroup analysis. In fact, nearly half (46.7%) of the patients in the placebo arm received open-label anlotinib, which may be a bias favourable for the placebo group. After adjustment, the median OS was 40.6 months versus 13.9 months for two groups (HR = 0.467 [95% CI, 0.222–0.980], *P* = 0.039).

The incidence of bone events is higher in patients with MTC than those with other types of thyroid cancer and is associated with impaired survival (16). The proportion of patients who had bone metastases was 51.6% in our study, which was higher than in ZETA (35.6%) and EXAM studies (50.9%) (6, 7). The risk of disease progression in patients with bone metastases was significantly reduced by 56% with anlotinib compared to placebo. There was also an increasing trend of OS benefit that the HR was 0.75 (95% CI, 0.28–2.01) and 0.67 (95% CI, 0.29–1.55) at the two timepoints of the analyses. Nine (50%) patients with bone metastases in the placebo arm received open-label anlotinib, which may partly explain the lack of statistical significance in OS prolongation.

In our study, although a high proportion (approximately 50%) of these patients in the placebo arm received open-label anlotinib after disease progression, the prognosis was still inferior compared to their counterparts in the anlotinib group, indicating the importance of early treatment. In terms of the significant survival benefit of anlotinib treatment, earlier treatment with MKIs for this population is necessary and meaningful. This view was also supported by the treatment of differentiated thyroid carcinoma (DTC) with anlotinib (17).

Long-term treatment with MKIs may be required for locally advanced or metastatic MTC. Therefore, toxicity often becomes a barrier to achieving maximal efficacy, since AEs can induce treatment interruption or termination. Anlotinib showed a favourable safety and tolerance profile in MTC, although the incidence of AEs could not be directly compared between different studies. In ALTER01031, the incidence of dose reduction was 32.2% with anlotinib in the entire population, whereas in previous studies, the incidence was 79% with cabozantinib and 35% with vandetanib (6, 7, 10). The incidence of treatment discontinuation and dose reduction of anlotinib in MTC seemed to be higher than that in other tumours (18, 19), which may be partly due to the prolonged duration of treatment. For example, in a study of anlotinib as second-line treatment for renal clear cell carcinoma (RCC), the incidence of dose adjustment was only 11.9% (median PFS: 8.5 months) (20). Accordingly, the incidence of dose adjustment for cabozantinib in the second-line treatment for RCC was 69% (median PFS: 7.4 months) (21), indicating that the favourable safety profile of anlotinib was consistent across various cancer types.

The poor physical condition of patients with older age and bone metastases may reduce treatment tolerability and result in a higher incidence of AE. In our study, 70.6% of patients with older age and 63.8% of patients with bone metastases had ECOG PS score of 1, which was higher than that of the entire population (61.5%). As was expected, the incidence of grade 3 or worse TRAE in patients who received placebo in the two subgroups was 53.3% and 50.0%, respectively, which was twice as high as that in the entire population (24.1%), indicating that patients with older age or bone metastases had a tendency for functional disorders and abnormal laboratory indices spontaneously. Surprisingly, the incidence of grade 3 or worse TRAEs in patients with poor prognostic factors in the anlotinib arm was similar to that in the placebo arm. In older patients, the incidence of grade 3 or worse TRAEs was 55.6% versus 53.3% (*P* = 0.884) in patients with bone metastases, and the incidence of grade 3 or worse TRAEs was 58.6% versus 50.0% (*P* = 0.563).

High-grade QT intervals or haemorrhage frequently limit the use of MKIs in older patients and those with bone metastases. For example, vandetanib and cabozantinib are not suitable for patients with heart conduction disorders or those with a higher risk of haemorrhage or fistula formation, respectively (6, 7). As we reported previously, anlotinib may be feasible for these populations, as no high-grade electrocardiogram QT interval prolongation and only one case of high-grade haemorrhage event were observed in this study.

Overall, in patients with metastatic MTC with older age or bone metastases, anlotinib still showed favourable safety and safety profiles. Particularly, a significant OS benefit was observed in older patients. These results suggest that intervention with MKIs, such as anlotinib, may be beneficial for patients with locally advanced or metastatic MTC with negative prognostic factors.

This study has some limitations. This was a subgroup analysis, and the results should be interpreted with caution. Compared with the ZETA and EXAM studies, the sample size of ALTER01031 was relatively small, and a phase III study is in process.

## Conclusion

Anlotinib demonstrated impressive survival benefits in patients with locally advanced or metastatic MTC with negative prognostic factors, including older age or bone metastases. The safety profile was consistent with that of the entire study population.

## Data availability statement

The data analyzed in this study is subject to the following licenses/restrictions: Some or all data, models, or code that support the findings of this study are available from the corresponding author upon reasonable request. Requests to access these datasets should be directed to anlozxq@126.com.

## Ethics statement

The studies involving human participants were reviewed and approved by ethics committee of Tianjin Medical University Cancer Institute and Hospital; Ethics committee of National Clinical Research Center for Cancer. The patients/participants provided their written informed consent to participate in this study.

## Author contributions

XZ and MG designed the study and were responsible for the conduction of study. JZZ, YihC, XC, MHG, YZ, ZG, JW, JC, JWZ, YinC, ZL, HL, JQ, JQZ, RC, ZX, and PT participated in the study and data collection. JZZ and YihC analyzed data and drafted the report. All authors reviewed and approved the submitted article.

## Funding

ALTER01031 was funded by the Chia Tai TianQing Pharmaceutical Group Co., Ltd. The funders had no role in the design of the study, the data collection, analysis, or the decision to publish the manuscript.

## Acknowledgments

We thank the patients participated in this study. We thank and for the assistance of data analysis. ALTER01031 was funded by the Chia Tai TianQing Pharmaceutical Group Co., Ltd.

## Conflict of interest

The authors declare that the research was conducted in the absence of any commercial or financial relationships that could be construed as a potential conflict of interest.

## Publisher’s note

All claims expressed in this article are solely those of the authors and do not necessarily represent those of their affiliated organizations, or those of the publisher, the editors and the reviewers. Any product that may be evaluated in this article, or claim that may be made by its manufacturer, is not guaranteed or endorsed by the publisher.

## References

[1] Lim H Devesa SS Sosa JA Check D Kitahara CM. Trends in thyroid cancer incidence and mortality in the united states, 1974-2013. JAMA (2017) 317:1338–48. doi: 10.1001/jama.2017.2719 PMC8216772 28362912

[2] Ceolin L Duval MADS Benini AF Ferreira CV Maia AL. Medullary thyroid carcinoma beyond surgery: advances, challenges, and perspectives. Endocr Relat Cancer (2019) 26:R499–518. doi: 10.1530/ERC-18-0574 31252403

[3] Martins RG Rajendran JG Capell P Byrd DR Mankoff DA. Medullary thyroid cancer: options for systemic therapy of metastatic disease? J Clin Oncol (2006) 24:1653–5. doi: 10.1200/JCO.2005.05.4106 16549818

[4] Ricarte-Filho JC Ryder M Chitale DA Rivera M Heguy A Ladanyi M. Mutational profile of advanced primary and metastatic radioactive iodine-refractory thyroid cancers reveals distinct pathogenetic roles for BRAF, PIK3CA, and AKT1. Cancer Res (2009) 69:69. doi: 10.1158/0008-5472.CAN-09-0727 PMC2690720 19487299

[5] Erovic BM Kim D Cassol C Goldstein DP Irish JC Asa SL. Prognostic and predictive markers in medullary thyroid carcinoma. Endocr Pathol (2012) 23:232–42. doi: 10.1007/s12022-012-9225-8 23150029

[6] Elisei R Schlumberger MJ Müller SP Schöffski P Brose MS Shah MH. Cabozantinib in progressive medullary thyroid cancer. J Clin Oncol (2013) 31:3639–46. doi: 10.1200/JCO.2012.48.4659 PMC4164813 24002501

[7] Wells SA Jr Robinson BG Gagel RF Dralle H Fagin JA Santoro M. Vandetanib in patients with locally advanced or metastatic medullary thyroid cancer: a randomized, double-blind phase III trial. J Clin Oncol (2012) 30:134–41. doi: 10.1200/JCO.2011.35.5040 PMC3675689 22025146

[8] Wirth LJ Sherman E Robinson B Solomon B Kang H Lorch J. Efficacy of selpercatinib in RET-altered thyroid cancers. N Engl J Med (2020) 383:825–35. doi: 10.1056/NEJMoa2005651 PMC10777663 32846061

[9] Hu M Subbiah V Wirth LJ Schuler M Taylor M. Results from the registrational phase i/ii arrow trial of pralsetinib (blu-667) in patients (pts) with advanced ret mutation-positive medullary thyroid cancer (ret+ mtc). Ann Oncol (2020) 31:S1084. doi: 10.1016/j.annonc.2020.08.1401

[10] Shen G Zheng F Ren D Du F Dong Q Wang Z. Anlotinib: a novel multi-targeting tyrosine kinase inhibitor in clinical development. J Hematol Oncol (2018) 11:120. doi: 10.1186/s13045-018-0664-7 30231931 PMC6146601

[11] Li D Chi Y Chen X Ge M Zhang Y Guo Z. Anlotinib in locally advanced or metastatic medullary thyroid carcinoma: A randomized, double-blind phase IIB trial. Clin Cancer Res (2021) 27:3567–75. doi: 10.1158/1078-0432.CCR-20-2950 33832949

[12] Sahli ZT Canner JK Zeiger MA Mathur A. Association between age and disease specific mortality in medullary thyroid cancer. Am J Surg (2021) 221:478–84. doi: 10.1016/j.amjsurg.2020.09.025 PMC9169693 33010878

[13] Xu JY Murphy WA Jr Milton DR Jimenez C Rao SN Habra MA. Bone metastases and skeletal-related events in medullary thyroid carcinoma. J Clin Endocrinol Metab (2016) 101:4871–7. doi: 10.1210/jc.2016-2815 PMC5155685 27662441

[14] Choksi P Papaleontiou M Guo C Worden F Banerjee M Haymart M. Skeletal complications and mortality in thyroid cancer: A population-based study. J Clin Endocrinol Metab (2017) 102:1254–60. doi: 10.1210/jc.2016-3906 PMC5460727 28324052

[15] Qu N Shi RL Luo TX Wang YL Li DS Wang Y. Prognostic significance and optimal cutoff of age in medullary thyroid cancer. Oncotarget (2016) 7:15937–47. doi: 10.18632/oncotarget.7556 PMC4941288 26910117

[16] Vogel T Wendler J Frank-Raue K Kreissl MC Spitzweg C Fassnacht M. Bone metastases in medullary thyroid carcinoma: High morbidity and poor prognosis associated with osteolytic morphology. J Clin Endocrinol Metab (2020) 105:dgaa077. doi: 10.1210/clinem/dgaa077 32072159

[17] Chi Y Gao M Zhang Y Shi F Cheng Y Guo Z. Anlotinib in radioiodine-refractory differentiated thyroid carcinoma: A subanalysis based on ALTER01032 study for patients with poor baseline characteristics. J Clin Oncol (2021) 39 suppl 15:6022 (abstract). doi: 10.1200/JCO.2021.39.15_suppl.6022

[18] Han B Li K Wang Q Zhang L Shi J Wang Z. Effect of anlotinib as a third-line or further treatment on overall survival of patients with advanced non-small cell lung cancer: The ALTER 0303 phase 3 randomized clinical trial. JAMA Oncol (2018) 4:1569–75. doi: 10.1001/jamaoncol.2018.3039 PMC6248083 30098152

[19] Zhou AP Bai Y Song Y Luo H Ren XB Wang X. Anlotinib versus sunitinib as first-line treatment for metastatic renal cell carcinoma: A randomized phase II clinical trial. Oncologist (2019) 24:e702–8. doi: 10.1634/theoncologist.2018-0839 PMC6693716 30902918

[20] Ma J Song Y Shou J Bai Y Li H Xie X. Anlotinib for patients with metastatic renal cell carcinoma previously treated with one vascular endothelial growth factor receptor-tyrosine kinase inhibitor: A phase 2 trial. Front Oncol (2020) 10:664. doi: 10.3389/fonc.2020.00664 32457838 PMC7221023

[21] Choueiri TK Escudier B Powles T Mainwaring PN Rini BI Donskov F. Cabozantinib versus everolimus in advanced renal-cell carcinoma. N Engl J Med (2015) 373:1814–23. doi: 10.1056/NEJMoa1510016 PMC5024539 26406150

